# Targeting proteostasis and autophagy in SMARCB1-deficient malignancies: where next?

**DOI:** 10.18632/oncotarget.26970

**Published:** 2019-06-18

**Authors:** Pavlos Msaouel, Alessandro Carugo, Giannicola Genovese

**Affiliations:** Department of Genitourinary Medical Oncology, The University of Texas MD Anderson Cancer Center, Houston, TX 77030, USA; David H. Koch Center for Applied Research of Genitourinary Cancers, The University of Texas MD Anderson Cancer Center, Houston, TX 77030, USA; Department of Genomic Medicine, The University of Texas MD Anderson Cancer Center, Houston, TX 77030, USA

**Keywords:** autophagy, malignant rhabdoid tumors, proteasome inhibitors, renal medullary carcinoma, SMARCB1

The SWItch/Sucrose Non-Fermentable (SWI/SNF) complex hydrolyzes adenosine triphosphate (ATP) to remodel chromatin structure. SWI/SNF complex defects occur in many types of cancer, with one or more SWI/SNF subunits mutated in ~20% of all human malignancies [1]. The inactivation of SMARCB1 (also known as INI1, hSNF5, or BAF47), a critical subunit of the SWI/SNF complex, deregulates the activity of SWI/SNF resulting in highly aggressive tumorigenesis. Conditional inactivation of the *Smarcb1* gene leads to cancer in 100% of mice with a median onset of only 11 weeks [2]. This high penetrance and rapid transformation is rarely observed with the inactivation of other single genes in cancer biology and underscores the potent tumor suppressor role of SMARCB1. Inactivation of SMARCB1 is found in all cases of renal medullary carcinoma (RMC) and renal cell carcinoma unclassified with medullary phenotype (RCCU-MP); in the majority of malignant rhabdoid tumors (MRT), atypical teratoid/rhabdoid tumors (ATRT), and epithelioid sarcomas (ES); and in aggressive variants of pancreatic carcinomas, extraskeletal myxoid chondrosarcomas, sinonasal carcinomas, and primitive neuroectodermal tumors [3–5]. Notably, the two prototypical SMARCB1-deficient malignancies, RMC and MRT, have very low mutation rates with SMARCB1 loss being the only recurrent event, suggesting that SMARCB1 inactivation is sufficient to drive these highly malignant tumors [3, 6]. However, there are currently no approved therapies directed toward SMARCB1 defects.

Although rare, RMC is the third most common renal cell carcinoma found in young patients and carries a dismal prognosis with <5% of patients surviving longer than 36 months despite best currently available therapies [5]. RMC predominantly afflicts young adults and adolescents (median age 28 years old) with sickle cell trait and other sickle hemoglobinopathies. RCCU-MP is a variant of RMC with similarly aggressive clinical behavior. The only established difference between the two entities is that RMC occurs in individuals with sickle hemoglobinopathies, including sickle cell trait, whereas the RCCU-MP variant develops in the absence of sickle hemoglobinopathies and is at least ten times less frequent than RMC [5]. The increased regional ischemia induced by red blood cell sickling in the medullary vasa recta of individuals with sickle hemoglobinopathies may predispose renal inner medulla cells to SMARCB1 loss resulting in the much higher incidence of RMC compared with RCCU-MP [4]. MRT and ATRT are aggressive malignancies, occurring mostly in children younger than 3 years old, for which no standard treatment has been established. SMARCB1 is also inactivated in ~90% of ES, a soft-tissue sarcoma with limited treatment options. Thus, therapies targeted against SMARCB1 loss can benefit multiple malignancies that are highly lethal and refractory to standard therapies.

**Figure 1:**
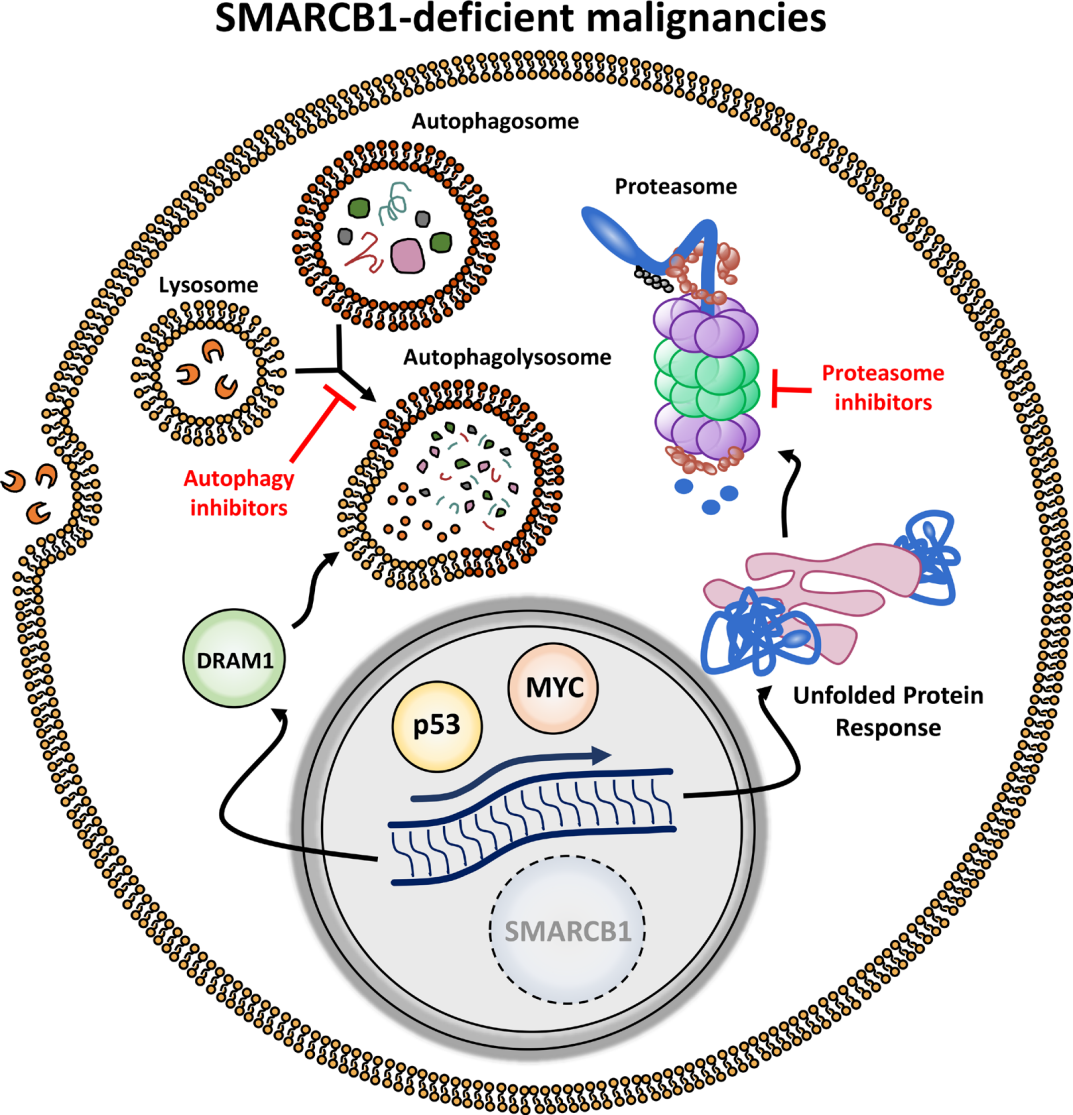
Targeting synthetic vulnerabilities induced by stress responses in SMARCB1-deficient malignancies. Loss of SMARCB1 induces upregulation of MYC and p53 resulting in increased proteotoxic stress making cells dependent on intact autophagy and unfolded protein response pathways. These pathways can be targeted by autophagy and proteasome inhibitors, respectively.

SMARCB1 loss profoundly activates the transcription factor MYC, resulting in significant upregulation of protein anabolism which can render cells susceptible to disruption of their proteostatic machinery [7, 8]. To further investigate the biological mechanisms underlying this finding, we developed *Smarcb1*-deficient embryonic mosaic mouse models of MRT and found that SMARCB1-deficient cancer cells show profound evidence of endoplasmic reticulum (ER) stress, including ER swelling, reticulophagy, alterations in the ER-ribosome interfase, and prominent accumulation of cytoplasmic protein aggregates. Cells adapt to this high stress by activating cellular programs regulated by the MYC-p19^ARF^-p53 axis and involved in protein disposal (proteasome pathway) and autophagy [8]. As a result, SMARCB1-deficient tumors become exquisitely sensitive to drugs that inhibit the proteasome and autophagic machineries (Figure 1). Indeed, we observed potent and durable responses of *in vitro* and *in vivo* MRT and RMC models treated with proteasome inhibitors, such as bortezomib and ixazomib, and/or the autophagy inhibitor chloroquine [8]. These findings were further validated in a separate study which noted that RMC cell lines are synthetically vulnerable to proteasome inhibitors [9].

Notably, one case report described a durable (>24 months) complete response to monotherapy with bortezomib in a patient with RMC, a disease that has to date been refractory to all other targeted therapies clinically tested. However, this result was not replicated in other patients with RMC who received single-agent bortezomib [5]. The clinical presentation of RMC is more reminiscent of aggressive hematological malignancies, which often require a combination of drugs to achieve potent and durable responses. Indeed, the combination of bortezomib with cytotoxic chemotherapy achieved gratifying and durable responses in two pediatric patients with RMC [5], thus providing clinical evidence that combining proteasome inhibition with chemotherapy should be further investigated. Novel regimens combining proteasome inhibitors with gemcitabine and doxorubicin have recently been developed for the treatment of urothelial carcinoma [10]. The combination of gemcitabine with doxorubicin is one of the most clinically active cytotoxic chemotherapy regimens used for the treatment of RMC. It is therefore an excellent cytotoxic backbone for testing whether the addition of proteasome inhibitors can improve the outcomes of patients with RMC, the most common SMARCB1-deficient renal cell carcinoma. Accordingly, we activated a phase II trial (NCT03587662 at clinicaltrials.gov) to evaluate the efficacy of ixazomib combined with gemcitabine and doxorubicin in ≥12 years old patients with aggressive SMARCB1-deficient kidney malignancies: RMC, RCCU-MP, and adult-onset kidney MRT. To our knowledge, this is the only ongoing clinical trial specifically targeting SMARCB1 loss.

Much work remains to be accomplished as SMARCB1-deficient malignancies are extremely aggressive and it is doubtful that a single regimen will help all patients and all diseases. Autophagy can protect patients from chemotherapy-induced kidney injury and from the cardiotoxicity of anthracyclines such as doxorubicin. Given the promising clinical reports with bortezomib and the rarity of these diseases, we chose to investigate first in the phase II setting the efficacy of proteasome inhibitors combined with cytotoxic chemotherapy, and to evaluate in future phase I/II trials the clinical synergy and toxicity of adding autophagy inhibitors. We are accordingly clinically evaluating potential biomarkers of autophagy regulation and sensitivity to the combination of ixazomib with cytotoxic chemotherapy. These insights may allow tailoring of targeted treatment combinations based on the specific adaptive mechanisms to proteotoxic stress (proteasome and/or autophagy pathways) used by SMARCB1-deficient tumors in each individual patient.
